# ID 335 - Priorização de Diretrizes Clínico-Assistenciais para o SUS: relato de experiência sobre a elaboração de uma revisão de escopo

**DOI:** 10.5327/2237-9622.2025.v34s1.335

**Published:** 2025-11-25

**Authors:** Maíra Barroso Pereira, Adriane Lopes Medeiros Simone, Tamiê de Camargo Martins, Andreia Ramos Lira, Tainá Freitas Saldanha, Rafaela Alves Padua, Guilherme de Almeida Moraes, Edwirgens Mariana Albuquerque, Gabrielli Mariano Fidêncio Casarini, Valter Antônio da Silva, Emilly Kelly Silva Monteiro, Bruna Cristina da Cruz Lima, Mônica Cristiane Rodrigues, Stéfani Sousa Borges, Daniela Oliveira de Melo

**Keywords:** diretrizes clínico-assistenciais, critérios, priorização, sistemas de saúde, avaliação de tecnologias em saúde

## Abstract

**Introdução:**

A Avaliação de Tecnologias em Saúde (ATS) é fundamental para orientar políticas de saúde com base em evidências. Um dos principais desafios enfrentados pelos gestores do Sistema Único de Saúde (SUS) na implementação da Política Nacional de Gestão de Tecnologias em Saúde (PNGTS) é consolidar critérios e processos claros para priorizar a incorporação de tecnologias e a atualização das diretrizes de cuidado. Esse desafio é resultado de uma atualização legislativa, que prevê que os Protocolos Clínicos e Diretrizes Terapêuticas (PCDT) sejam atualizados periodicamente, à luz de novos estudos e evidências científicas. Nesse contexto, a construção de uma diretriz metodológica de sistematização do processo de priorização é oportuna. Inicialmente, foi realizada uma revisão de escopo (RE) para mapear modelos e critérios nacionais e internacionais de priorização de temas para diretrizes clínico-assistenciais (DCA) em sistemas e serviços de saúde. Este relato de experiência tem o objetivo de descrever o processo de elaboração da RE e compartilhar as lições aprendidas.

**Método:**

A RE foi realizada entre janeiro e agosto de 2024, com a participação de 12 pesquisadores do grupo de pesquisa Chronide do Núcleo de Avaliação de Tecnologias em Saúde da Universidade Federal de São Paulo, campus Diadema (Unifesp-Diadema). Após um período de capacitação para o alinhamento da metodologia, a RE foi desenvolvida segundo metodologia proposta no do Joanna Briggs Institute e em conformidade com a checklist da extensão do para RE. O protocolo foi registrado no .

**Resultados:**

A questão da revisão foi delineada em: população, conceito e contexto. Foram incluídos estudos primários e secundários, sem restrição de idioma e publicados a partir de 2000. Estabeleceu-se a estratégia de busca pelos descritores de cada base de dados, como MEDLINE, Embase e Biblioteca Virtual em Saúde (BVS), além de buscas complementares. Após retirada das duplicatas, 17.666 publicações foram avaliadas por título e resumo por pares de pesquisadores independentes, no software Rayyan®. Na etapa de leitura de texto completo, 32 referências foram identificadas elegíveis para a extração e a análise dos dados. Dessas publicações, 87,5% são artigos, publicados de 2017 a 2024 (62,5%), predominantemente europeus (40,6%). Das publicações, 56,3% relataram não utilizar métodos de consenso e 59,4% não tratam de intervenções específicas.

**Conclusão:**

A amplitude da questão da revisão gerou uma grande quantidade de estudos para análise, resultado de uma estratégia de busca bem delineada para diferentes bases de dados. Esse desafio, somado à repetição do exercício de seleção, durante a capacitação da equipe com diferentes níveis de formação, fortaleceu a comunicação e a colaboração entre os pesquisadores, resultando em uma visão mais coesa e compartilhada do escopo, que refletirá na qualidade da diretriz metodológica. A utilização do Rayyan® facilitou a organização e a análise dos dados, tornando o processo mais eficiente. A extração dos dados demandou maior esforço devido às diferenças nos delineamentos dos estudos e à variabilidade dos dados, exigindo gestão de tempo, flexibilidade e adaptabilidade da equipe. Conclui-se que esta iniciativa, ao agregar ensino-pesquisa-extensão, fortalece a PNGTS, impulsionando a formação de recursos humanos para a Rede Brasileira de Avaliação de Tecnologias em Saúde (Rebrats), e também amplia o impacto social da formação universitária, pois seu produto contribuirá para suprir necessidades do SUS.

